# Air Pollution Exposure and Markers of Placental Growth and Function: The Generation R Study

**DOI:** 10.1289/ehp.1204918

**Published:** 2012-08-24

**Authors:** Edith H. van den Hooven, Frank H. Pierik, Yvonne de Kluizenaar, Albert Hofman, Sjoerd W. van Ratingen, Peter Y.J. Zandveld, Henk Russcher, Jan Lindemans, Henk M.E. Miedema, Eric A.P. Steegers, Vincent W.V. Jaddoe

**Affiliations:** 1 The Generation R Study Group, Erasmus Medical Center, Rotterdam, the Netherlands; 2 Urban Environment and Safety, TNO (Netherlands Organization for Applied Scientific Research), Utrecht, the Netherlands; 3 Department of Epidemiology,; 4 Department of Clinical Chemistry,; 5 Department of Obstetrics and Gynaecology, and; 6 Department of Paediatrics, Erasmus Medical Center, Rotterdam, the Netherlands

**Keywords:** air pollution, dispersion modeling, nitrogen dioxide, particulate matter, placenta, pregnancy

## Abstract

Background: Air pollution exposure during pregnancy might affect placental growth and function, perhaps leading to pregnancy complications.

Objective: We prospectively evaluated the associations of maternal air pollution exposure with markers of placental growth and function among 7,801 pregnant women in the Netherlands.

Methods: We estimated levels of particulate matter ≤ 10 µm in aerodynamic diameter (PM_10_) and nitrogen dioxide (NO_2_) at the home address for different periods during pregnancy using dispersion modeling techniques. Pro- and anti-angiogenic factors [placental growth factor (PlGF) and soluble fms-like tyrosine kinase 1 (sFlt-1), respectively] were measured in first- and second-trimester maternal blood and in fetal cord blood samples at delivery. Pulsatility index of the uterine and umbilical arteries was measured by Doppler ultrasound in second and third trimester, and notching was assessed in third trimester. Placenta weight and birth weight were obtained from medical records.

Results: Higher PM_10_ and NO_2_ exposure levels were associated with lower second-trimester maternal sFlt-1 and PlGF levels. PM_10_ and NO_2_ exposures averaged over total pregnancy were associated with higher sFlt-1 and lower PlGF levels in fetal cord blood, consistent with an anti-angiogenic state. PM_10_ and NO_2_ exposures were not consistently associated with second- or third-trimester placental resistance indices. NO_2_ exposure was associated with third-trimester notching (odds ratio 1.33; 95% CI: 0.99, 1.78 per 10-µg/m^3^ increase in the prior 2 months). PM_10_ and NO_2_ exposures were associated with lower placenta weight (–11.8 g; 95% CI: –20.9, –2.7, and –10.7 g; 95% CI: –19.0, –2.4, respectively, per 10-µg/m^3^ increase in the prior 2 months), but not with placenta to birth weight ratio.

Conclusions: Our results suggest that maternal air pollution exposure may influence markers of placental growth and function. Future studies are needed to confirm these findings and explore the maternal and fetal consequences.

Air pollution exposure during pregnancy has been associated with fetal growth restriction, low birth weight, and preterm birth (Bonzini et al. 2010; Shah et al. 2011; van den Hooven et al. 2012b). Previous studies have also reported associations of maternal air pollution exposure with preeclampsia and gestational hypertension (van den Hooven et al. 2011; Wu et al. 2009). These maternal and fetal complications are suggested to have their origin largely in abnormal early placentation, which is characterized by impaired tropho-blast invasion and lack of modification of the spiral arteries (Kaufmann et al. 2003; Ness and Sibai 2006; Schlembach et al. 2007; Steegers et al. 2010). As a result, the arteries maintain a higher vascular resistance, which could eventually lead to impaired uteroplacental perfusion and development of maternal and fetal complications (Kaufmann et al. 2003; Ness and Sibai 2006; Schlembach et al. 2007; Steegers et al. 2010).

Maternal air pollution exposure may affect pregnancy by inducing oxidative stress and systemic inflammation (Brook et al. 2010), which could result in suboptimal placentation or placental inflammation (Dejmek et al. 1999; Kannan et al. 2006; van den Hooven et al. 2012a), and subsequent development of fetal growth restriction and maternal hypertensive complications. Various markers of placental growth and function can be studied in relation to the development of maternal and fetal complications. The angiogenic growth factors vascular endothelial growth factor (VEGF) and placental growth factor (PlGF) are important for placental development and angiogenesis, whereas soluble fms-like tyrosine kinase 1 (sFlt-1) binds to these proteins and thereby inhibits their activity. Previous studies have demonstrated elevated blood sFlt-1 levels or reduced blood PlGF levels in women whose pregnancies were complicated by intrauterine growth restriction, preeclampsia, and gestational hypertension (Asvold et al. 2011; Levine et al. 2004; Smith et al. 2007; Thadhani et al. 2004), and in infants of mothers with preeclampsia (Catarino et al. 2009; Staff et al. 2005); but other studies reported no associations or associations in the opposite direction (Asvold et al. 2011; Jacobs et al. 2011; Smith et al. 2007; Thadhani et al. 2004; Widmer et al. 2007). In addition, indices of placental vascular resistance and the presence of uterine artery notching (an abnormality of the Doppler ultrasound waveform that indicates increased blood flow resistance) have been used to identify complicated pregnancies (Cnossen et al. 2008; Schlembach et al. 2007). In normal pregnancy, the pulsatility index of the uterine arteries decreases with advancing gestational age, as a result of the trophoblast invasion during the first half of pregnancy. Impaired remodeling of the arteries leads to maintenance of high arterial resistance, with subsequent inadequate uteroplacental blood flow (Kaufmann et al. 2003). Furthermore, the placental ratio (placenta weight relative to birth weight) can be considered a marker of placental function—the capacity to transport oxygen and nutrients. A high placental ratio is suggested to reflect a less efficient placental function and reduced nutrient supply to the fetus (Barker et al. 1993; Salafia et al. 2006). A low placenta weight and a high placental ratio have been linked to adverse pregnancy outcomes in various studies (Lao and Wong 1996; Mayhew et al. 2003).

If suboptimal placental function contributes to associations between air pollution and pregnancy complications, underlying mechanisms may include effects of air pollution on angiogenesis, placental vascular resistance, and placental growth. Therefore, we investigated associations of maternal exposure to particulate matter ≤ 10 µm in aerodynamic diameter (PM_10_) and nitrogen dioxide (NO_2_) during pregnancy with maternal and fetal angiogenic factors, placental vascular resistance indices, and placenta weight in a population-based cohort study among 7,801 pregnant women living in an urban area in the Netherlands.

## Methods

*Design.* This study was embedded in the Generation R Study, a population-based prospective cohort study from early pregnancy onward in the city of Rotterdam, the Netherlands (Jaddoe et al. 2010). Mothers were enrolled between 2001 and 2005. The study protocol was approved by the Medical Ethical Committee of Erasmus Medical Center, Rotterdam. All participants provided written informed consent. Of the 8,880 prenatally enrolled women, air pollution exposure estimates were available for 7,914 mothers (89%). For 966 mothers, air pollution exposure data could not be assessed because of incomplete address history, or because they had moved outside the study area before delivery (Jaddoe et al. 2010). Mothers with a twin pregnancy (*n* = 84), abortion (*n* = 7), or intrauterine death (*n* = 12) were excluded. Of the mothers with singleton live births, 10 mothers were excluded because of missing data for angiogenic factors, placental vascular resistance, or placenta weight. Associations between air pollution exposure and markers of placental growth and function were analyzed in the remaining 7,801 participants [see Supplemental Material, Figure S1, for a flow chart and exact numbers for each analysis, and Supplemental Material, Figure S2, for the timing of the different measurements (http://dx.doi.org/10.1289/ehp.1204918)].

*Air pollution exposure.* Individual exposures to PM_10_ and NO_2_ during pregnancy were assessed at the home address, using a combination of continuous monitoring data and dispersion modeling techniques, taking into account both the spatial and temporal variation in air pollution. The method has been previously described in detail (van den Hooven et al. 2011, 2012c). In brief, annual average concentrations of PM_10_ and NO_2_ for the years 2001–2006 were estimated for all addresses in the study area, using the three Dutch national standard methods for air quality modeling (Netherlands Ministry of Infrastructure and the Environment 2007). The performance of this modeling procedure based on (a combination of) the three standard methods has been evaluated by two previous studies in the same study area. These studies reported a good agreement between predicted annual average PM_10_ and NO_2_ concentrations and concentrations measured at monitoring stations (Beelen et al. 2010; Keuken et al. 2011). Next, hourly concentrations of PM_10_ and NO_2_ were derived for each address, taking into account hourly wind conditions and fixed temporal patterns in the contribution of air pollution sources. Subsequently, the hourly concentrations were adjusted for background concentrations, using hourly measurements from three continuous monitoring stations. We obtained full residential histories for participants, which showed that 13% of the women moved at least once during pregnancy. Based on participants’ home addresses, we derived individual exposure estimates for different periods preceding the outcome measurements, to examine the effects of both short-term exposures (2 weeks before the outcome measurement) and longer-term exposures (2 months before, and averaged over the pregnancy period from conception until outcome measurement).

*Angiogenic factors.* Maternal nonfasting venous blood samples were collected in first trimester (median, 13.2 weeks; 95% range: 9.5–17.5) and second trimester (median, 20.4 weeks; 95% range: 18.5–23.5) [see Supplemental Material, Figure S2 (http://dx.doi.org/10.1289/ehp.1204918]. Midwives and obstetricians sampled umbilical venous cord blood immediately after delivery (median gestational age at delivery, 40.1 weeks; 95% range: 35.4–42.3). All blood samples were transported to the regional laboratory for processing and storage at –80^o^C (Jaddoe et al. 2007). Concentrations of sFlt-1 and PlGF were measured in EDTA plasma samples at the Department of Clinical Chemistry of the Erasmus Medical Center between 2008 and 2010, using a two-step chemi-luminescent microparticle immunoassay (CMIA) technology on the Architect System (Abbot Diagnostics B.V., Hoofddorp, the Netherlands). The between-run coefficients of variation for plasma sFlt-1 were 2.8% at 5.5 ng/mL and 2.3% at 34.0 ng/mL, and the coefficients for plasma PlGF were 4.7% at 24 pg/mL, and 3.8% at 113 pg/mL. The highest level of detection was 150 ng/mL for sFlt-1 and 1,500 pg/mL for PlGF.

*Placental vascular resistance*. Placental vascular resistance was evaluated with flow velocity waveforms from the uterine and umbilical arteries in second trimester (median, 20.5 weeks; 95% range: 18.7–23.3) and third trimester (median, 30.3 weeks; 95% range: 28.4–32.9) [see Supplemental Material, Figure S2 (http://dx.doi.org/10.1289/ehp.1204918]. Raised uterine and umbilical artery pulsatility indices indicate increased uteroplacental and fetoplacental resistances, respectively (Miller et al. 2008). Uterine artery pulsatility index was measured in the right and left uterine artery near the crossover with the external iliac artery, and the mean value was calculated. The presence of unilateral or bilateral uterine artery notching was assessed in the third trimester. Umbilical artery pulsatility index was measured in a free-floating loop of the umbilical cord. For each measurement three consecutive uniform waveforms were recorded by pulsed Doppler ultrasound, during fetal apnea and without fetal movement. The mean of three measurements was used for further analysis.

*Placenta weight and placental ratio*. Information on placenta weight and birth weight was obtained from medical records completed by midwives and obstetricians. Each placenta was weighed fresh, with membrane and umbilical cord attached, within one hour after delivery. Placental ratio was calculated as (placenta weight/birth weight) × 100%.

*Covariates*. Information on date of delivery, gestational age at delivery, and infant sex was obtained from medical records. Information on maternal age, parity, educational level, ethnicity, and folic acid supplementation use was obtained by a questionnaire at enrolment (median gestational age, 14.4 weeks; 95% range: 10.2–29.5). Because there were no differences in observed results when ethnicity was categorized into five groups instead of two groups, we reclassified ethnicity as European or non-European. Maternal anthropometrics were assessed at time of enrolment. Maternal smoking and alcohol consumption before and during pregnancy were assessed by questionnaires in each trimester, and were categorized as none, first trimester only, or continued during pregnancy. Month of conception and month of birth were categorized into seasons: winter (December–February), spring (March –May), summer (June–August), and fall (September–November). Road traffic noise exposure (*L_den_*) at the home address at delivery was assessed in accordance with requirements of the Environmental Noise Directive (European Commission 2002). The assessment procedure has been described previously (de Kluizenaar et al. 2007; van den Hooven et al. 2011).

*Statistical analysis*. First, maternal (first and second trimester) and fetal sFlt-1 and PlGF levels were log-transformed (using the natural log) to obtain normally distributed outcome variables. To prevent the introduction of missing values in transformed variables, concentrations of 0 ng/mL for fetal sFlt-1 (*n* = 29; 0.8%) and 0 pg/mL for fetal PlGF (*n* = 154; 0.1%) were imputed by random draws from the left tail of a normal distribution (Helsel 2005) (corresponding to values < 0.024 ng/mL and < 3.5 pg/mL, respectively). Imputing these values resulted in marginal changes in *p*-values and point estimates for the associations between air pollution and fetal sFlt-1 and PlGF levels, which was probably attributable to larger numbers. Next, we used linear regression models to estimate associations between air pollution exposures and maternal and fetal sFlt-1 and PlGF levels. We report coefficients for log-transformed concentrations multiplied by 100, which can be interpreted as percentage changes (Cole 2000). Furthermore, we used linear regression models to estimate associations of air pollution exposures with placental vascular resistance indices (in SD values, calculated as resistance index/SD of the resistance index in the population), placenta weight, birth weight, and placental ratio, and used logistic regression models to estimate associations with third trimester notching. For all linear regression models, we first checked the linearity of the associations by plotting the standardized residuals against the predicted residuals before we performed the analyses. All models were adjusted for known determinants of pregnancy outcomes, determined *a priori* (Coolman et al. 2012; Sibai et al. 2005), including maternal age, body mass index, parity, ethnicity, education, smoking, alcohol consumption, folic acid supplementation use, gestational age at measurement, and infant sex (coded as continuous or categorical variables; Table 1). In addition, we evaluated the influence of noise exposure, season of conception, and maternal height on effect estimates. Based on a 10% change in effect estimates, we adjusted models of placenta weight, birth weight, and placental ratio for noise exposure and season of conception, and adjusted models of birth weight for maternal height. As additional sensitivity analyses, we restricted analyses to nonsmoking women, European women, nonobese women, and women without pregnancy complications (gestational hypertension, preeclampsia, or gestational diabetes), and also performed a sensitivity analysis additionally adjusting for season of birth. The percentages of missing values within the population for analysis were < 1% for continuous data and < 15% for all categorical variables except folic acid supplementation use, which was missing for 26% of observations. To reduce bias associated with missing data, covariates were multiple imputed (*n* = 5 imputations) based on the correlation between the variable with missing values and other participant characteristics (Sterne et al. 2009). Data were imputed according to the Markov Chain Monte Carlo method, because no monotone missing pattern was found. Detailed information on the imputation procedure and population characteristics based on the original data set and the imputed data sets are presented in Supplemental Material, Tables S1 and S2 (http://dx.doi.org/10.1289/ehp.1204918). Analyses were performed using both the original data set and the imputed data sets. Because we observed similar effect estimates, we present only the pooled effect estimates (differences and odds ratios) with their 95% CIs after the multiple imputation procedure. All statistical analyses were performed using PASW version 17.0 for Windows (PASW Inc., Chicago, IL, USA). *p*-Values of < 0.05 were considered statistically significant.

**Table 1:** Participant characteristics (*n* = 7,801).

Maternal characteristics	Mean ± SD, median (95% range), or *n* (%)
Age at enrollment (years)	30.3 (19.2–39.3)
Gestational age at enrollment (weeks)	14.4 (10.2–29.5)
Height (cm)	167.1 ± 7.5
Weight (kg)	67.0 (50.0–103.0)
Body mass index at enrolment (kg/m2)	23.8 (18.7–36.3)
Parity
Nulliparous	4,290 (55.6)
Multiparous	3,420 (44.4)
Missing	91
Ethnic background
European	4,140 (57.3)
Non-European	3,088 (42.7)
Missing	573
Highest completed educational level
No education/primary	814 (11.5)
Secondary	3,219 (45.3)
Higher	3,071 (43.3)
Missing	697
Smoking in pregnancy
No	4,987 (74.0)
First trimester only	574 (8.5)
Continued	1,174 (17.4)
Missing	1,066
Alcohol consumption in pregnancy
No	3,295 (48.5)
First trimester only	905 (13.3)
Continued	2,592 (38.2)
Missing	1,009
Folic acid supplementation use
Preconceptional	2,340 (40.5)
First 10 weeks of pregnancy	1,793 (31.0)
None	1,679 (29.1)
Missing	2,025
Season of conception
Winter	2,198 (28.2)
Spring	1,794 (23.0)
Summer	1,772 (22.7)
Fall	2,037 (26.1)
Noise exposure based on home address at delivery [dB(A)]	52.7 (45.0–68.2)
Of the total group, data were missing on maternal height (n = 28), maternal weight (n = 35), body mass index at enrollment (n = 62), and noise exposure (n = 159).		

## Results

*Participant and exposure characteristics.* Table 1 presents the maternal characteristics. The median age of the participants was 30.3 years. Most women were nulliparous, and 43.3% had completed higher education. Table 2 presents the outcome characteristics and shows that median maternal sFlt-1 levels were stable in first and second trimester, whereas median PlGF levels increased from first to second trimester. As expected, uterine and umbilical artery pulsatility indices decreased throughout gestation. Mean (± SD) placenta weight was 635 ± 146 g, and mean offspring birth weight was 3,414 ± 559 g. The number of participants with available exposure and outcome data per ana-lysis varied in our study. However, we did not observe major differences in characteristics between the eligible participants for each analysis (results not shown). Mean exposure levels during pregnancy were 30.3 µg/m^3^ for PM_10_ and 39.9 µg/m^3^ for NO_2_ [see Supplemental Material, Table S3, for descriptive data on air pollution exposures by trimester and time period before measurement (http://dx.doi.org/10.1289/ehp.1204918]. Pearson correlation coefficients between the different exposure averages varied between 0.25 and 0.93 (data not shown). Correlations among exposure averages for the prior 2 weeks and 2 months were moderate to strong (PM_10_: *r* = 0.58 to 0.61; NO_2_: *r* = 0.78 to 0.80).

**Table 2:** Outcome characteristics.

Characteristic	Mean ± SD, median (95% range), or n (%)
Angiogenic factors
First trimester, gestational age at visit (weeks) (n = 5,043)	13.2 (9.6–17.5)
Maternal sFlt-1 (ng/ml)	5.1 (1.9–14.3)
Maternal PlGF (pg/ml)	42.2 (14.6–188.4)
Second trimester, gestational age at visit (weeks) (n = 6,368)	20.6 (18.5–23.5)
Maternal sFlt-1 (ng/ml)	5.0 (1.5–17.4)
Maternal PlGF (pg/ml)	201.2 (73.8–623.7)
Delivery, gestational age (weeks) (n = 3,667)	40.1 (36.6–42.3)
Fetal sFlt-1 (ng/ml)	0.5 (0.1–5.9)
Fetal PlGF (pg/ml)	8.6 (0.0–21.9)
Placental vascular resistance
Second trimester, gestational age at visit (weeks) (n = 5,510)	20.5 (18.7–23.3)
Uterine artery pulsatility index	0.90 ± 0.27
Umbilical artery pulsatility index	1.20 ± 0.19
Third trimester, gestational age at visit (weeks) (n = 6,080)	30.3 (28.4–32.9)
Uterine artery pulsatility index	0.74 ± 0.19
Umbilical artery pulsatility index	0.98 ± 0.17
Presence of unilateral uterine artery notching	303 (6.8)
Presence of bilateral uterine artery notching	141 (3.2)
Birth characteristics
Gestational age at birth (weeks) (n = 7,688)	40.1 (35.6–42.3)
Placenta weight (g)	635 ± 146
Birth weight (g)	3,414 ± 559
Placental ratio (%)	18.7 ± 3.5

*Air pollution and angiogenic factors*. PM_10_ and NO_2_ exposures in different periods (i.e., the prior 2 weeks, prior 2 months, and from the time of conception to outcome measurement) were not significantly associated with first-trimester maternal sFlt-1 levels, but were associated with lower second-trimester sFlt-1 levels (differences, –4.3%; 95% CI: –7.4, –1.1%; and –2.7%; 95% CI: –5.1, –0.2% per 10-µg/m^3^ increase in PM_10_ and NO_2_ in the prior 2 months, respectively) (Table 3-). In contrast, PM_10_ and NO_2_ exposures averaged over total pregnancy were associated with higher fetal sFlt-1 levels at delivery (differences, 35.8%; 95% CI: 25.6, 45.9%; and 8.9%; 95% CI: 0.6, 17.3 per 10-µg/m^3^ increase in PM_10_ and NO_2_, respectively). Average PM_10_ exposure during the prior 2 months and from the time of conception was associated with significantly higher maternal PlGF levels in first trimester, but with nonsignificant lower PlGF levels in second trimester. Average NO_2_ exposure during the prior 2 months and from the time of conception was associated with significantly lower PlGF levels in second trimester. Inverse associations were observed for PM_10_ and NO_2_ exposure for the prior 2 months and during total pregnancy with fetal PlGF levels at delivery (differences, –16.3%; 95% CI: –21.9, –10.7; and –14.6%; 95% CI: –19.3, –10.0 for PM_10_ and NO_2_ during total pregnancy, respectively). The unadjusted estimates generally were consistent with the adjusted estimates, although negative associations between NO_2_ and second-trimester sFlt-1 and PlGF levels were weaker before adjustment [see Supplemental Material, Table S4 (http://dx.doi.org/10.1289/ehp.1204918)].

**Table 3:** Associations of maternal air pollution exposure with percent changes in angiogenic factors in first and second trimester and at delivery [percent change (95% CI)].

Air pollution exposure	Maternal sFlt-1		Fetal sFlt-1	Maternal PlGF		Fetal PlGF
	First trimester (n = 4,993)	Second trimester (n = 6,365)	Delivery (n = 3,629)	First trimester (n = 5,024)	Second trimester (n = 6,365)	Delivery (n = 3,224)
PM10 (per 10 µg/m3)
Prior 2 weeks	0.1 (–1.7, 2.0)	–2.7 (–4.7, –0.7)*	–0.6 (–5.2, 3.9)	0.0 (–1.6, 1.7)	–1.5 (–3.1, 0.2)#	–0.5 (–3.1, 2.1)
Prior 2 months	–0.2 (–3.1, 2.7)	–4.3 (–7.4, –1.1)*	–2.8 (–10.1, 4.4)	3.0 (0.4, 5.6)*	–1.9 (–4.5, 0.7)	–14.4 (–18.3, –10.5)**
Total pregnancy perioda	1.1 (–2.3, 4.6)	–4.5 (–8.4, –0.5)*	35.8 (25.6, 45.9)**	3.6 (0.5, 6.7)*	–1.1 (–4.3, 2.1)	–16.3 (–21.9, –10.7)**
NO2 (per 10 µg/m3)
Prior 2 weeks	1.3 (–0.5, 3.1)	–1.8 (–3.7, 0.1)#	5.0 (0.6, 9.5)*	0.5 (–1.1, 2.1)	–1.0 (–2.6, 0.5)	–4.1 (–6.5, –1.8)*
Prior 2 months	0.7 (–1.7, 3.0)	–2.7 (–5.1, –0.2)*	3.4 (–2.0, 8.9)	0.4 (–1.7, 2.5)	–2.7 (–4.7, –0.7)*	–10.4 (–13.3, –7.5)**
Total pregnancy perioda	0.7 (–2.0, 3.4)	–2.1 (–5.1, 1.0)	8.9 (0.6, 17.3)*	0.2 (–2.2, 2.6)	–2.8 (–5.3, –0.3)*	–14.6 (–19.3, –10.0)**
Values are regression coefficients and reflect the percent change (95% range) in log-transformed soluble fms-like tyrosine kinase 1 (sFlt-1) and placental growth factor (PlGF) levels per 10‑µg/m3 increase in air pollution exposure. Models are adjusted for gestational age at measurement, fetal sex, maternal age, body mass index, parity, ethnicity, education, smoking, alcohol consumption, and folic acid supplementation use. aAir pollution exposure for the total pregnancy period was estimated as average exposure for the period from conception until first-trimester measurement, from conception until second-trimester measurement, or from conception until delivery. **p < 0.001, *p < 0.05, #p < 0.10.												

*Air pollution and placental vascular resistance*. We did not observe consistent associations of air pollution exposures with uterine and umbilical artery pulsatility indices (Table 4). Average PM_10_ exposure during the prior 2 months and from the time of conception was associated with a significantly lower umbilical artery pulsatility index in the second trimester (differences, –0.07 SD; 95% CI: –0.13, –0.02; and –0.10 SD; 95% CI: –0.17, –0.03 per 10-µg/m^3^ increase, respectively). Unadjusted model estimates were similar [see Supplemental Material, Table S5 (http://dx.doi.org/10.1289/ehp.1204918)]. PM_10_ and NO_2_ exposure during the prior 2 weeks and prior 2 months were positively but not significantly associated with bilateral uterine artery notching [e.g., for NO_2_, odds ratio (OR) = 1.22; 95% CI: 0.97, 1.53; and OR = 1.33; 95% CI: 0.99, 1.78, respectively) (Table 5). Unadjusted estimates suggested slightly stronger associations for NO_2_ exposure and bilateral notching, but were otherwise similar to adjusted estimates (see Supplemental Material, Table S6).

**Table 4:** Associations of maternal air pollution exposure with uteroplacental and fetoplacental vascular resistance in second and third trimester [difference (95% CI)].

Air pollution exposure	Uterine artery pulsatility index (SD)		Umbilical artery pulsatility index (SD)	
	Second trimester (n = 3,432)	Third trimester (n = 3,511)	Second trimester (n = 5,443)	Third trimester (n = 6,026)
PM10 (per 10 µg/m3)
Prior 2 weeks	0.00 (–0.05, 0.05)	–0.03 (–0.08, 0.02)	–0.02 (–0.06, 0.01)	0.01 (–0.03, 0.04)
Prior 2 months	0.03 (–0.05, 0.11)	–0.01 (–0.08, 0.07)	–0.07 (–0.13, –0.02)*	0.04 (–0.02, 0.10)
Total pregnancy perioda	0.02 (–0.08, 0.11)	–0.06 (–0.17, 0.04)	–0.10 (–0.17, –0.03)*	–0.01 (–0.09, 0.06)
NO2 (per 10 µg/m3)
Prior 2 weeks	0.01 (–0.03, 0.06)	–0.03 (–0.07, 0.02)	–0.01 (–0.04, 0.02)	–0.01 (–0.04, 0.02)
Prior 2 months	0.02 (–0.04, 0.08)	–0.02 (–0.07, 0.03)	–0.02 (–0.06, 0.02)	0.03 (–0.01, 0.07)
Total pregnancy perioda	0.02 (–0.05, 0.09)	–0.04 (–0.11, 0.04)	–0.04 (–0.09, 0.01)	0.03 (–0.03, 0.08)
Values are regression coefficients and reflect the difference in SD score of uterine and umbilical artery pulsatility index per 10‑µg/m3 increase in air pollution exposure. Models are adjusted for gestational age at measurement, fetal sex, maternal age, body mass index, parity, ethnicity, educational level, smoking, alcohol consumption, and folic acid supplementation use. aAir pollution exposure for the total pregnancy period was estimated as average exposure for the period from conception until second-trimester measurement or from conception until third-trimester measurement. *p < 0.05.								

**Table 5:** Associations of maternal air pollution exposure with uterine artery notching in third trimester (95% CI).

Air pollution exposure	Unilateral notching odds ratio (n = 4,244)	Bilateral notching odds ratio (n = 4,091)
PM10 (per 10 µg/m3)
Prior 2 weeks	0.94 (0.77, 1.12)	1.18 (0.92, 1.51)
Prior 2 months	0.93 (0.70, 1.23)	1.31 (0.88, 1.94)
Total pregnancy perioda	0.96 (0.66, 1.38)	1.14 (0.67, 1.93)
NO2 (per 10 µg/m3)
Prior 2 weeks	0.99 (0.85, 1.16)	1.22 (0.97, 1.53)#
Prior 2 months	0.96 (0.79, 1.16)	1.33 (0.99, 1.78)#
Total pregnancy perioda	1.12 (0.85, 1.48)	1.18 (0.79, 1.76)
Values are odds ratios and reflect the risk for unilateral and bilateral uterine artery notching in third-trimester per 10‑µg/m3 increase in air pollution exposure. Models are adjusted for gestational age at measurement, fetal sex, maternal age, body mass index, parity, ethnicity, educational level, smoking, alcohol consumption, and folic acid supplementation use. aAir pollution exposure for the total pregnancy period was estimated as average exposure for the period from conception until third-trimester measurement. #p < 0.10.				

*Air pollution and placenta weight*. PM_10_ and NO_2_ exposure during the 2 months preceding delivery were associated with a lower placenta weight (differences, –11.8g; 95% CI: –20.9, –2.7; and –10.7; 95% CI: –19.0, –2.4 per 10-µg/m^3^ increase, respectively), but no significant associations were observed for exposure during the prior 2 weeks or over the entire pregnancy (Table 6). PM_10_ and NO_2_ exposures during all three time periods were associated with significant reductions in birth weight (differences, –34.6; 95% CI: –66.3, –2.9; and –39.3; 95% CI: –69.1, –9.6 per 10-µg/m^3^ increase in PM_10_ and NO_2_ during total pregnancy, respectively). PM_10_ exposure for the prior 2 months was associated with a reduced placental ratio, but no significant associations were observed for exposure during other time periods or for NO_2_ exposure. Unadjusted models showed similar results, although associations of PM_10_ and NO_2_ exposure during the 2 months preceding delivery with placenta weight were weaker [see Supplemental Material, Table S7 (http://dx.doi.org/10.1289/ehp.1204918)].

**Table 6:** Associations of maternal air pollution exposure with placenta weight, birth weight, and placental ratio (95% CI).

Air pollution exposure	Placenta weight (g) difference (n = 5,605)	Birth weight (g) difference (n = 7,688)	Placental ratio (%) difference (n = 5,599)
PM10 (per 10 µg/m3)
Prior 2 weeks	–1.7 (–7.0, 3.6)	–16.0 (–29.5, –2.4)*	0.0 (–0.1, 0.2)
Prior 2 months	–11.8 (–20.9, –2.7)*	–37.9 (–61.4, –14.4)*	–0.2 (–0.5, 0.0)*
Total pregnancy	–6.0 (–18.5, 6.4)	–34.6 (–66.3, –2.9)*	–0.1 (–0.4, 0.2)
NO2 (per 10 µg/m3)
Prior 2 weeks	–2.8 (–8.8, 3.2)	–17.1 (–32.4, –1.8)*	0.1 (–0.1, 0.2)
Prior 2 months	–10.7 (–19.0, –2.4)*	–24.3 (–45.6, –3.1)*	–0.2 (–0.4, 0.0)
Total pregnancy	–9.3 (–20.9, 2.3)	–39.3 (–69.1, –9.6)*	–0.1 (–0.4, 0.1)
Values are regression coefficients and reflect the difference in placenta weight, birth weight, and placental ratio [(placenta weight/birth weight) × 100%] per 10-µg/m3 increase in air pollution exposure. Models are adjusted for gestational age at delivery, infant sex, maternal age, body mass index, parity, ethnicity, educational level, smoking, alcohol consumption, folic acid supplementation use, noise exposure, and season of conception. Models on birth weight are additionally adjusted for maternal height. *p < 0.05.						

*Sensitivity analyses*. Results in nonsmoking women were comparable to those for the cohort as a whole (data not shown). Associations were slightly stronger when the analyses were restricted to European women or women with a normal body mass index, but the patterns of associations were the same (data not shown). Restriction to women without pregnancy complications (gestational hypertension, preeclampsia, or gestational diabetes) produced similar results, although associations between NO_2_ exposure and bilateral uterine artery notching in the third trimester were weaker and nonsignificant (e.g., for the prior 2 months, OR = 1.24; 95% CI: 0.90, 1.71; data not shown). When we additionally adjusted the analyses for season of birth, results were comparable (data not shown).

## Discussion

Results from this large prospective cohort study suggest that maternal air pollution exposure may affect placental growth and function. We observed associations of PM_10_ and NO_2_ exposure with changes in fetal sFlt-1 and PlGF levels at delivery. Also, higher PM_10_ and NO_2_ exposures were associated with lower placenta weight. However, air pollution exposure was not consistently associated with other markers of placental growth and function.

*Air pollution and markers of placental function and growth*. Adequate placentation and placental functioning are critical for normal pregnancy. Impairment of these processes, reflected by alterations in markers of placental growth and function, has been associated with maternal and fetal complications (Levine et al. 2004; Ness and Sibai 2006; Salafia et al. 2006; Schlembach et al. 2007). Maternal air pollution exposure has been hypothesized to affect placentation and placental function (Dejmek et al. 1999; Kannan et al. 2006), but associations between air pollution exposures and markers of placental growth and function have rarely been studied.

Over the past decades there has been increased interest in the role of angiogenic growth factors in the development of maternal and fetal complications. PlGF is considered to be important for placental development and angiogenesis, whereas sFlt-1 binds to PlGF and thereby inhibits its activity (Coolman et al. 2012). To our knowledge, no previous studies have examined the associations of air pollution with angiogenic factors during pregnancy. Our results regarding sFlt-1 and PlGF levels were contradictory. We observed that PM_10_ and NO_2_ exposure were associated with lower maternal second-trimester sFlt-1 levels, and weakly associated with higher first-trimester PlGF levels and lower second-trimester PlGF levels. At delivery, averaged air pollution exposure over total pregnancy was associated with higher fetal sFlt-1 levels and lower PlGF levels. These cord blood levels might reflect placental rather than fetal production (Schlembach et al. 2007; Staff et al. 2005). Thus, results suggest that air pollution may have contributed to an anti-angiogenic profile (i.e., increased sFlt-1 and decreased PlGF levels) in fetal cord blood, but not in maternal first- and second-trimester blood. In this light, it is interesting that maternal smoking, which shares similarities in biological effects with air pollution, was associated with lower sFlt-1 levels and higher PlGF levels (i.e., a pro-angiogenic profile) in maternal first- and second-trimester blood (e.g., for continued smoking, –8.3%; 95% CI: –13.3, –3.2% for sFlt-1 levels in the first trimester and 27.1%; 95% CI: 21.8, 32.4% for PlGF levels in the first trimester). Previous studies on maternal smoking during pregnancy have also reported lower sFlt-1 levels, indicating that smoking could promote a pro-angiogenic balance. Our associations of air pollution with angiogenic factors differed according to the trimester of pregnancy, which may reflect trimester-specific alterations in sFlt-1 and PlGF levels in response to the pregnancy (Hirashima et al. 2005). Also, it has been proposed that elevated sFlt-1 levels could be attributable to increased trophoblastic placental tissue (Bdolah et al. 2008), which may indicate that increased levels of sFlt-1 and PlGF could merely reflect an increase in trophoblast volume rather than pathological processes.

Compared with uncomplicated pregnancies, complicated pregnancies are more often characterized by a relatively high placental vascular resistance and the presence of uterine artery notching, an additional indicator of increased placental vascular resistance (Cnossen et al. 2008; Schlembach et al. 2007). Overall, we observed no consistent associations of air pollution exposure with uterine or umbilical artery pulsatility indices in second and third trimester. However, average PM_10_ exposure during the prior 2 months and from the time of conception was associated with a reduced umbilical artery pulsatility index in second trimester. Because we hypothesized that air pollution would adversely affect placental function, this observation was inconsistent with our expectations. Furthermore, the results are also not consistent with previous studies on maternal smoking that reported increased uteroplacental and fetoplacental resistance in smokers compared with nonsmoking mothers (Geelhoed et al. 2011; Machado Jde et al. 2011). However, in our study NO_2_ exposure in the period preceding the measurement was associated with nonsignificant increased ORs for bilateral uterine artery notching, an indication of increased arterial resistance that is an established risk factor for maternal and fetal complications (Cnossen et al. 2008; Kaufmann et al. 2003). The clinical relevance of the time- and pollutant-specific associations observed between air pollution exposure and placental vascular resistance in our study population needs further study.

A low placenta weight and a high placental ratio (placenta weight relative to birth weight) may reflect less efficient placental function (Barker et al. 1993; Salafia et al. 2006). Previous experimental studies in mice have reported associations of air pollution exposure during pregnancy with lower placenta weight (Rocha e Silva et al. 2008) and with morphological changes of the placenta that suggested impaired placental function (Veras et al. 2008). Two previous studies examined the associations of (indicators of) air pollution exposure with placenta weight or ratio in women. The first study was conducted in Italy and reported a decreased placenta weight following exposure to PM_10_ and NO_2_ in the two months before delivery (differences –3 and –7 g per 10-µg/m^3^ increase in PM_10_ and NO_2_, respectively) (Pesatori et al. 2008). In the second study, conducted in Japan, living within 200 m of a major road (as an indicator of air pollution exposure) was associated with a 13-g decrease in placenta weight and a 0.5% increase in placental ratio, compared with living farther away (Yorifuji et al. 2012). In the present study, we observed that PM_10_ and NO_2_ exposure in the 2 months preceding delivery were associated with reductions in placenta weight of 12 and 11 g per 10-µg/m^3^ increase, respectively—consistent with the Italian study. We previously reported that PM_10_ and NO_2_ exposures during different time periods were strongly associated with reductions in birth weight (van den Hooven et al. 2012b). We did not observe consistent associations of air pollution exposure with placental ratio in the present study, which may indicate that placenta weight and birth weight both were reduced to a similar degree by air pollution.

*Methodological considerations*. A main strength of our study is the availability of different markers of placental growth and function, enabling investigation of this possible pathway for air pollution effects on pregnancy outcomes. Information on a wide range of potential confounders also was available. Nevertheless, residual confounding due to unmeasured variables (e.g., quality of housing, including insulation and ventilation possibilities) might still be an issue.

Previous studies on air pollution exposure and markers of placental function and growth are limited. We considered a number of exposure windows to cover short- and longer-term exposures: 2 weeks and 2 months before outcome measurement, and from the time of conception to outcome measurement. We had initially examined other exposure windows (e.g., 1 week and 1 month prior) as well, but we did not present these results because the patterns of associations were similar to the presented findings. An important limitation of using overlapping exposure periods is that no conclusions can be drawn about the specific critical window of exposure. Future studies could examine the relevant exposure windows in more detail.

Blood samples were stored for a few years before sFlt-1 and PlGF concentrations were measured. It has been shown that PlGF and sFlt-1 levels are stable for at least 3 years when stored at –80°C (Law et al. 2010). Because of careful storage and processing procedures, we consider it unlikely that the storage time of the samples has affected our measurements. However, this possibility can not be excluded.

Air pollution is a complex mixture of several pollutants. PM_10_ and NO_2_ can be regarded as indicators of this mixture rather than the definite causative factors of adverse health effects. Air pollution exposure was estimated using a combination of dispersion modeling and continuous monitoring, which enabled consideration of detailed spatial and temporal variation in exposure. Incorporating temporal variation in exposure is often a concern with air pollution modeling. We performed a sensitivity analysis with additional adjustment for season of birth, which showed comparable results. This suggests that misclassification of the temporal adjustment was probably small in our study. The variation in exposure levels might be relatively small in our study population (e.g., a 10-µg/m^3^ increase in PM_10_ and NO_2_ exposure was larger than the interquartile range in different time periods), which may have limited our ability to detect associations of air pollution with markers of placental growth and function. Exposure estimates accounted for residential mobility of the women during pregnancy, but some exposure misclassification may have occurred because exposure levels were estimated for the home address only. No information was available on indoor, occupational, or commuting sources of air pollution, or on time–activity patterns of the women. A recent Canadian study showed that pregnant women spent more time at home than nonpregnant women, especially in the last stage of pregnancy (Nethery et al. 2009). If this is also true for Dutch pregnant women, the magnitude of the possible misclassification is probably less in our study population than it would be in a population of nonpregnant women.

## Conclusion

In this prospective population-based cohort study, maternal PM_10_ and NO_2_ exposure were associated with changes in fetal sFlt-1 and PlGF levels at delivery that are consistent with an anti-angiogenic state. This pattern was not observed for maternal sFlt-1 and PlGF in the first and second trimester of pregnancy. Also, maternal PM_10_ and NO_2_ exposure levels in the prior 2 months, but not in other exposure periods, were associated with lower placenta weight. Our results suggest that air pollution exposure may influence placental growth and function. Future studies are needed to confirm these findings, to examine the underlying mechanisms, and to explore the maternal and fetal consequences.

## Supplemental Material

(291 KB) PDF
