# Supplementary material for: Air Pollution Exposure and Markers of Placental Growth and Function: The Generation R Study
Source: Environ Health Perspect. 2012 Aug 24;120(12):1753–9. doi: 10.1289/ehp.1204918 (PMC3548279; doi:10.1289/ehp.1204918)
Supplement: (291 KB) PDF [file ehp.1204918.s001.pdf]

Supplemental Material

Air Pollution Exposure and Markers of Placental Growth and Function: The Generation R Study

Edith H. van den Hooven, Frank H Pierik, Yvonne de Kluizenaar, Albert Hofman, Sjoerd W. van Ratingen, Peter Y.J. Zandveld, Henk Russcher, Jan Lindemans, Henk M.E. Miedema, Eric A.P. Steegers, Vincent W.V. Jaddoe

Table of Contents

2 Figure S1. Population for analysis

3 Figure S2. Timing of measurements of markers of placental growth and function

4 Table S1. Details of the multiple imputation procedure

5 Table S2. Participant characteristics in the original dataset and the imputed dataset

7 Table S3. Distribution of PM_{10} and NO_2 exposure levels for different periods

9 Table S4. Unadjusted associations of maternal air pollution exposure with percent changes in angiogenic factors in first and second trimester and at delivery

10 Table S5. Unadjusted associations of maternal air pollution exposure with uteroplacental and fetoplacental vascular resistance in second and third trimester

11 Table S6. Unadjusted associations of maternal air pollution exposure with uterine artery notching in third trimester

12 Table S7. Unadjusted associations of maternal air pollution exposure with placenta weight, birth weight, and placental ratio

Supplemental Material, Figure S1. Population for analysis.

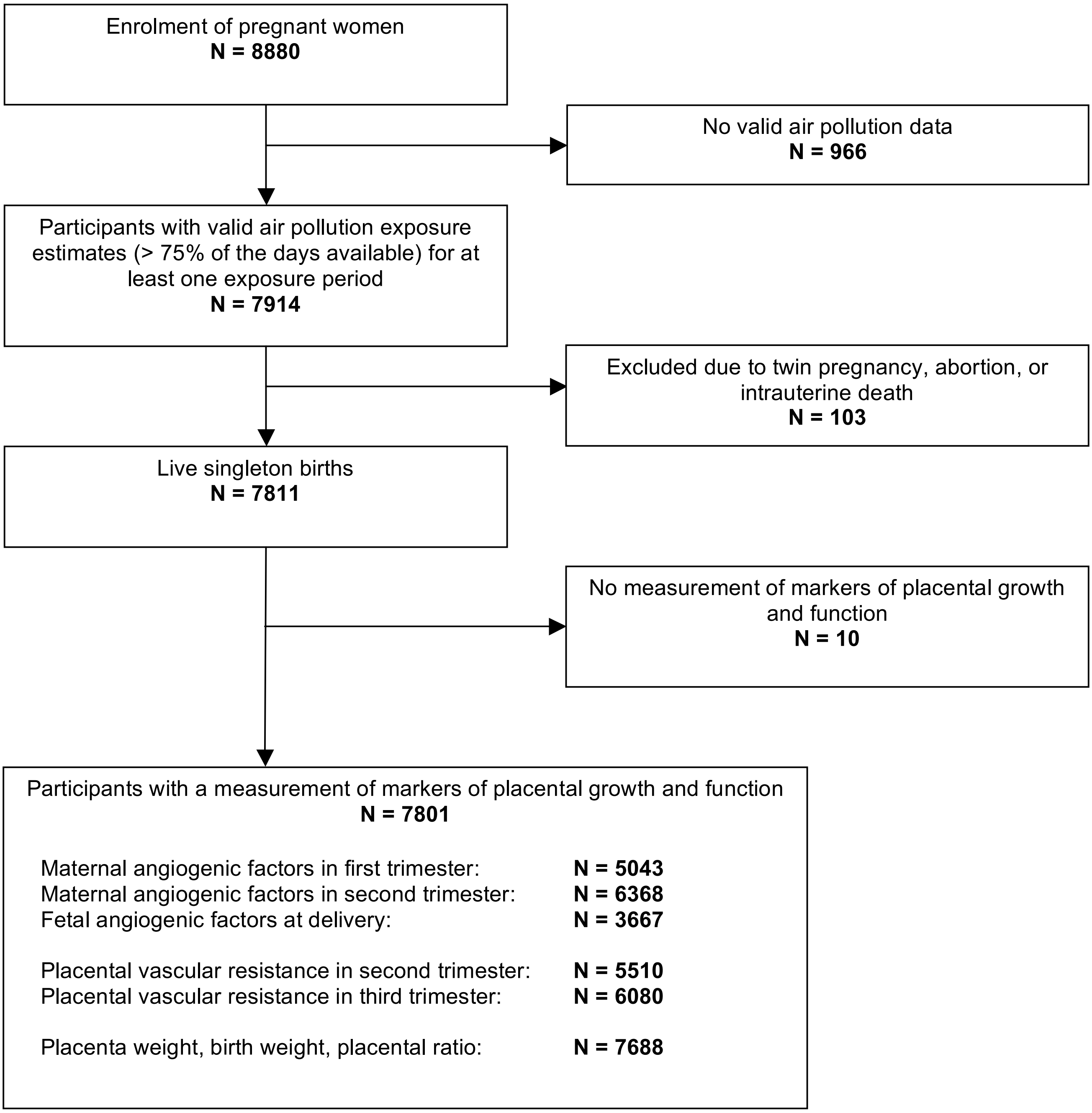

Supplemental Material, Figure S2. Timing of measurements of angiogenic factors, placental vascular resistance, and placenta weight.

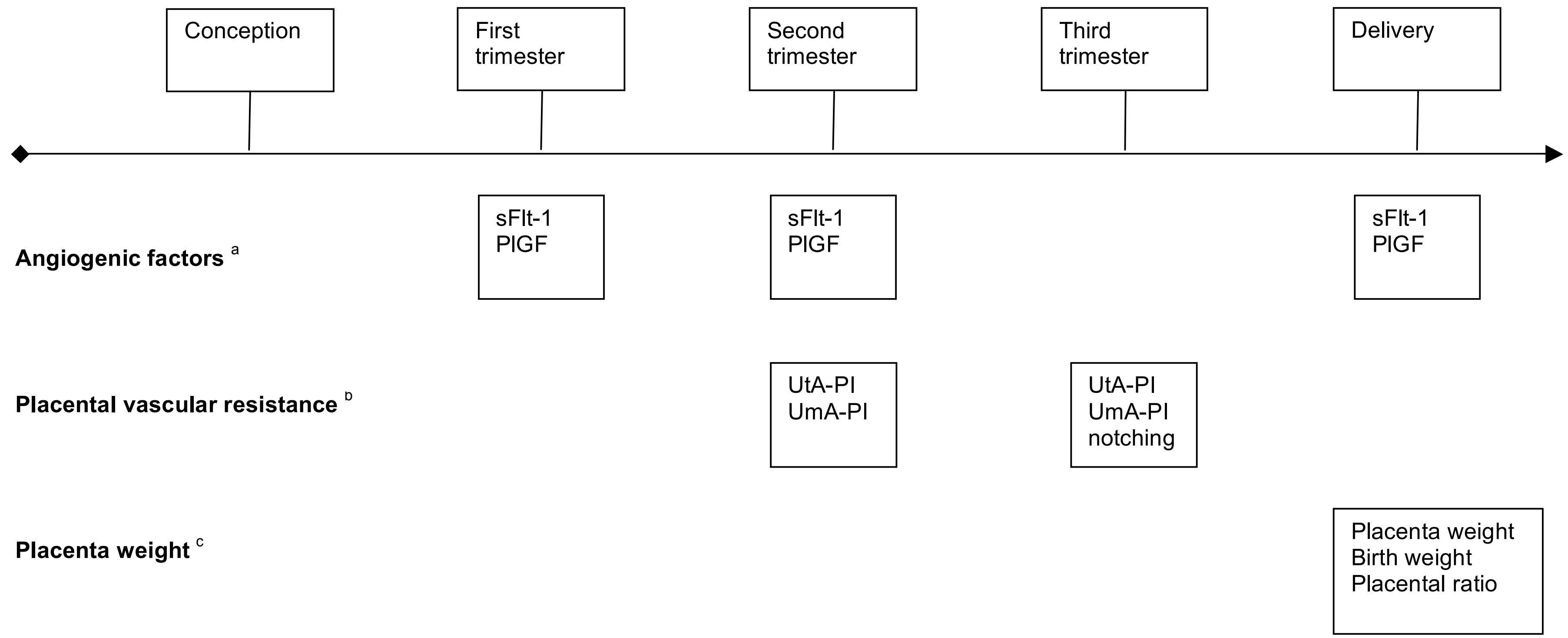

[a] Soluble fms-like tyrosine kinase 1 (sFlt-1) and placental growth factor (PlGF) were measured in maternal blood in first trimester (median 13.2 weeks of gestation, 95% range 9.6 to 17.5) and second trimester (median 20.6 weeks of gestation, 95% range 18.5 to 23.5), and in fetal cord blood at delivery (median 40.1 weeks of gestation, 95% range 36.6 to 42.3).

[b] Uterine and umbilical artery pulsatility index were measured in second trimester (median 20.5 weeks of gestation, 95% range 18.7 to 23.3) and third trimester (median 30.3 weeks of gestation, 95% range 28.4 to 32.9), and unilateral or bilateral uterine artery notching was assessed in third trimester.

[c] Placenta weight and birth weight were measured at delivery (median 40.1 weeks of gestation, 95% range 35.7 to 42.4), and placental ratio was calculated.

Supplemental Material, Table S1. Details of the multiple imputation procedure.

	Multiple imputation procedure
Sofware used:	PASW SPSS 17.0 for Windows
Imputation method and keysettings:	Fully conditional specification (Markov chain Monte Carlo method); Maximum iterations: 10
Number of imputed data sets created:	5
Variables included in the imputation procedure and used in main analyses, but not imputed (only used as predictors of missing data, since there were no missings):	Maternal age, gestational age at enrolment, fetal sex
Variable included in the imputation procedure and used in main analyses (imputed and used as predictors of missing data):	Maternal height, body mass index at enrolment, parity, ethnicity, education, smoking, alcohol consumption, folic acid supplementation use, gestational age at the outcome measurements, noise exposure
Variables not used in main analyses but used as predictors of missing data to increase plausibility of missing at random assumption:	Maternal weight at enrolment, average neighbourhood income, marital status, highest followed educational level, birth weight, SDS birth weight, household income, pre-pregnancy weight, pre-pregnancy body mass index, paternal height, paternal body mass index, paternal educational level
Treatment of non-normally distributed variables:	Log-transformed
Treatment of binary/categorical variables (i.e., maternal parity, ethnicity, education, marital status, smoking, alcohol consumption, folic acid supplementation use, highest followed educational level, average neighbourhood income, household income, paternal educational level):	Logistic regression

Supplemental Material, Table S2. Participant characteristics in the original dataset and the imputed datasets.

		Original dataset (observed percentage)	Imputed datasets (pooled results)
		mean ± SD, median (95% range), or percentage	mean ± SD, median (95% range), or percentage
**Maternal characteristics**			
Age at enrolment (yr)		30.3 (19.2-39.3)	30.3 (19.2-39.3)
	Missing - N	-	-
Gestational age at enrolment (weeks)		14.4 (10.2-29.5)	14.4 (10.2-29.5)
	Missing - N	-	-
Height (cm)		167.1 ± 7.5	167.1 ± 7.6
	Missing - N	28	-
Weight (kg)		67.0 (50.0-103.0)	69.4 (50.0-104.0)
	Missing - N	35	-
Body mass index (kg/m^2)		23.8 (18.7-36.3)	24.9 (18.6-36.5)
	Missing - N	62	-
Parity			
	Nulliparous	55.6%	55.6%
	Multiparous	44.4%	44.4%
	Missing - N	91	-
Ethnicity			
	European	57.3%	55.0%
	Non-European	42.7%	45.0%
	Missing - N	573	-
Educational level			
	No education/primary	11.5%	12.3%
	Secondary	45.3%	46.6%
	Higher	43.3%	41.0%
	Missing - N	697	-
Smoking in pregnancy			
	No	74.0%	74.0%
	First trimester only	8.5%	9.1%
	Continued	17.4%	16.8%
	Missing - N	1066	-

Supplemental Material, Table S2 (continued).

	**Original dataset (observed percentage)**	**Imputed datasets (pooled results)**
	mean ± SD, median (95% range), or percentage	mean ± SD, median (95% range), or percentage
**Maternal characteristics**		
Alcohol consumption in pregnancy		
No	48.5%	45.7%
First trimester only	13.3%	16.6%
Continued	38.2%	38.6%
Missing - N	1009	-
Folic acid supplementation use		
Preconceptional	40.5%	37.2%
First ten weeks of pregnancy	31.0%	30.7%
None	29.1%	32.1%
Missing - N	2025	-
Noise exposure based on home address at delivery (dB(A))	52.7 (45.0-68.2)	52.7 (45.0-68.2)
Missing - N	159	-
**Birth characteristics**		
Gestational age at birth (weeks)	39.8 (35.6-42.3)	39.8 (35.6-42.3)
Missing - N	3	-
Gender		
Male	50.7%	50.7%
Female	49.3%	49.3%
Missing - N	-	-

Values are means ± SDs, or medians (95% range) for variables with a skewed distribution, and percentages for categorical variables.

Supplemental Material, Table S3. Distribution of PM_{10} and NO_2 exposure levels for different periods.

	Minimum	25th percentile	Mean	Median	75th percentile	Maximum
**First trimester** [a]						
**PM_{10} exposure** ($\mu g/m^3$)						
Prior day 1-14	18.8	25.4	30.6	28.8	33.7	57.4
Prior day 1-60	21.4	26.9	30.7	30.5	33.6	46.2
Total pregnancy period [a]	22.0	27.7	30.9	30.8	33.7	44.0
**NO_2 exposure** ($\mu g/m^3$)						
Prior day 1-14	16.9	35.2	40.4	40.5	45.2	70.5
Prior day 1-60	20.2	36.7	40.4	41.0	44.1	59.3
Total pregnancy period [a]	21.0	37.2	40.5	41.0	44.0	59.8
**Second trimester** [b]						
**PM_{10} exposure** ($\mu g/m^3$)						
Prior day 1-14	17.4	25.0	30.3	28.7	33.3	56.4
Prior day 1-60	21.5	26.5	30.5	30.3	33.3	48.1
Total pregnancy period [b]	22.6	27.9	30.6	30.5	33.4	43.2
**NO_2 exposure** ($\mu g/m^3$)						
Prior day 1-14	15.1	34.6	40.2	40.2	45.0	66.9
Prior day 1-60	20.3	36.0	40.0	40.8	44.0	59.6
Total pregnancy period [b]	22.7	37.0	40.2	40.4	43.5	59.3
**Third trimester** [c]						
**PM_{10} exposure** ($\mu g/m^3$)						
Prior day 1-14	18.8	25.4	30.6	28.8	33.7	58.0
Prior day 1-60	20.2	26.3	30.6	29.4	33.8	49.5
Total pregnancy period [c]	22.7	27.5	30.0	30.0	32.4	41.5
**NO_2 exposure** ($\mu g/m^3$)						
Prior day 1-14	16.9	35.2	40.4	40.5	45.3	67.4
Prior day 1-60	19.8	35.8	40.4	40.8	44.5	65.5
Total pregnancy period [c]	25.6	36.8	39.8	39.6	42.5	58.2

Supplemental Material, Table S3 (continued).

	Minimum	25th percentile	Mean	Median	75th percentile	Maximum
**Delivery**						
**PM_{10} exposure** (µg/m3)						
Prior day 1-14	16.8	24.7	29.6	28.1	32.2	58.0
Prior day 1-60	21.4	26.1	29.7	29.5	32.4	45.8
Total pregnancy period	23.2	27.8	30.3	30.0	32.9	40.9
**NO_2 exposure** (µg/m3)						
Prior day 1-14	15.3	34.1	39.5	39.9	44.7	67.2
Prior day 1-60	18.5	35.1	39.4	40.3	43.6	65.3
Total pregnancy period	26.5	37.2	39.9	39.6	42.2	59.3

Air pollution exposure was estimated for two weeks (day 1-14) and two months (day 1-60) preceding the different measurements. Additionally, air pollution exposure was averaged over the period from the time of conception to outcome measurement (referred to as total pregnancy period).

[a] Air pollution exposure averages were calculated prior to the day of blood sampling (for measurement of angiogenic factors) in first trimester.

[b] Air pollution exposure averages were calculated prior to the day of blood sampling (for measurement of angiogenic factors) in second trimester. The ultrasound visit in second trimester was planned on the same day in the majority of the women. The corresponding exposure averages prior to this visit are not shown in the table.

[c] Air pollution exposure averages were calculated prior to the day of the ultrasound visit in third trimester.

Supplemental Material, Table S4. Unadjusted associations of maternal air pollution exposure with percent changes in angiogenic factors in first and second trimester and at delivery.

	**Maternal sFlt-1** Percent change (95% CI)		**Fetal sFlt-1** Percent change (95% CI)	**Maternal PlGF** Percent change (95% CI)		**Fetal PlGF** Percent change (95% CI)
	**First trimester** N=4993	**Second trimester** N=6365	**Delivery** N=3629	**First trimester** N=5024	**Second trimester** N=6365	**Delivery** N=3224
**PM_{10}** (per 10 μg/m^3)						
Prior two weeks	0.2 (-1.7, 2.1)	-2.2 (-4.3, -0.2) *	-0.6 (-5.1, 4.0)	0.2 (-1.5, 1.9)	-1.1 (-2.8, 0.6)	-0.5 (-3.1, 2.1)
Prior two months	-0.3 (-3.3, 2.7)	-3.7 (-6.9, -0.4) *	-2.4 (-9.7, 4.8)	3.7 (1.0, 6.4) *	-0.8 (-3.4, 1.9)	-14.6 (-18.5, -10.7) **
Total pregnancy period [a]	1.4 (-2.1, 4.9)	-3.5 (-7.6, 0.5) ‡	35.9 (25.8, 46.0) **	4.4 (1.2, 7.6) *	0.1 (-3.3, 3.5)	-16.6 (-22.2, -10.9) **
**NO_2** (per 10 μg/m^3)						
Prior two weeks	1.9 (0.0, 3.7) *	-0.7 (-2.7, 1.3)	5.4 (1.0, 9.8) *	1.0 (-0.7, 2.6)	-0.1 (-1.7, 1.5)	-4.3 (-6.7, -2.0) **
Prior two months	1.6 (-0.8, 4.0)	-0.8 (-3.4, 1.7)	3.9 (-1.6, 9.3)	1.2 (-1.0, 3.4)	-0.9 (-0.3, 1.2)	-10.7 (-13.6, -7.8) **
Total pregnancy period [a]	2.2 (-0.5, 4.9)	0.7 (-2.4, 3.8)	9.8 (1.6, 18.1) *	1.2 (-1.3, 3.6)	-0.5 (-3.0, 2.1)	-15.5 (-20.2, -10.9) **

** p < 0.001
* p < 0.05
‡ p < 0.10

Values are regression coefficients and reflect the percent change (95% range) in log-transformed soluble fms-like tyrosine kinase 1 (sFlt-1) and placental growth factor (PlGF) levels per 10 μg/m^3 increase in air pollution exposure. Models are adjusted for gestational age at measurement.

[a] Air pollution exposure for the total pregnancy period was estimated as average exposure for the period from conception until first trimester measurement, from conception until second trimester measurement, or from conception until delivery.

Supplemental Material, Table S5. Unadjusted associations of maternal air pollution exposure with uteroplacental and fetoplacental vascular resistance in second and third trimester.

	**Uterine artery Pulsatility Index** (SD) Difference (95% CI)		**Umbilical artery Pulsatility Index** (SD) Difference (95% CI)	
	**Second trimester** N=3432	**Third trimester** N=3511	**Second trimester** N=5443	**Third trimester** N=6026
**PM_{10}** (per 10 μg/m^3)				
Prior two weeks	0.00 (-0.05, 0.06)	-0.04 (-0.09, -0.02)	-0.02 (-0.06, 0.02)	0.01 (-0.03, 0.04)
Prior two months	0.03 (-0.05, 0.11)	-0.01 (-0.09, 0.07)	-0.06 (-0.12, -0.01) *	0.04 (-0.02, 0.10)
Total pregnancy period [a]	0.02 (-0.08, 0.11)	-0.08 (-0.18, 0.03)	-0.08 (-0.15, -0.01) *	-0.01 (-0.09, 0.07)
**NO_2** (per 10 μg/m^3)				
Prior two weeks	0.02 (-0.03, 0.06)	-0.03 (-0.07, 0.01)	-0.01 (-0.04, 0.03)	-0.01 (-0.02, 0.01)
Prior two months	0.03 (-0.03, 0.09)	-0.03 (-0.08, 0.03)	-0.01 (-0.05, 0.03)	0.03 (-0.01, 0.07)
Total pregnancy period [a]	0.03 (-0.04, 0.10)	-0.05 (-0.12, 0.03)	-0.02 (-0.07, 0.03)	0.03 (-0.02, 0.09)

* $p < 0.05$

Values are regression coefficients and reflect the difference in SD score of uterine and umbilical artery pulsatility index per 10 μg/m^3 increase in air pollution exposure. Models are adjusted for gestational age at measurement.

[a] Air pollution exposure for the total pregnancy period was estimated as average exposure for the period from conception until second trimester measurement or from conception until third trimester measurement.

Supplemental Material, Table S6. Unadjusted associations of maternal air pollution exposure with uterine artery notching in third trimester.

	**Unilateral notching**<br>Odds ratio (95% CI)<br>N=4244	**Bilateral notching**<br>Odds ratio (95% CI)<br>N=4091
**PM_{10}** (per 10 μg/m^3)		
Prior two weeks	0.93 (0.77, 1.12)	1.17 (0.92, 1.49)
Prior two months	0.94 (0.71, 1.25)	1.32 (0.89, 1.94)
Total pregnancy period	0.96 (0.67, 1.38)	1.14 (0.68, 1.92)
**NO_2** (per 10 μg/m^3)		
Prior two weeks	1.02 (0.87, 1.19)	1.27 (1.02, 1.58) *
Prior two months	1.00 (0.82, 1.21)	1.39 (1.04, 1.84) *
Total pregnancy period	0.98 (0.87, 1.10)	1.34 (0.92, 1.95)

* $p < 0.05$

Values are odds ratios and reflect the risks for unilateral and bilateral uterine artery notching in third trimester per 10 μg/m^3 increase in air pollution exposure. Models are adjusted for gestational age at measurement.

Air pollution exposure for the total pregnancy period was estimated as average exposure for the period from conception until third trimester measurement.

Supplemental Material, Table S7. Unadjusted associations of maternal air pollution exposure with placenta weight, birth weight, and placental ratio.

	**Placenta weight** (g) Difference (95% CI) N=5605	**Birth weight** (g) Difference (95% CI) N=7688	**Placental ratio** (%) Difference (95% CI) N=5599
**PM_{10}** (per 10 μg/m^3)			
Prior two weeks	-0.6 (-5.8, 4.6)	-13.8 (-28.2, 0.5) ‡	0.1 (-0.1, 0.2)
Prior two months	-8.3 (-16.8, 0.2) ‡	-36.9 (-60.3, -13.4) *	-0.2 (-0.4, 0.0)
Total pregnancy	-7.4 (-19.5, 4.7)	-54.0 (-87.0, -20.9) *	0.0 (-0.3, 0.3)
**NO_2** (per 10 μg/m^3)			
Prior two weeks	-1.0 (-5.9, 3.8)	-14.2 (-27.2, -1.2) *	0.0 (-0.1, 0.2)
Prior two months	-2.6 (-8.5, 3.3)	-16.4 (-32.4, -0.5) *	0.0 (-0.2, 0.1)
Total pregnancy	-5.6 (-14.8, 3.5)	-55.4 (-80.5, -30.3) **	0.1 (-0.1, 0.3)

* $p < 0.05$

‡ $p < 0.10$

Values are regression coefficients and reflect the difference in placenta weight, birth weight, and placental ratio ((placenta weight/birth weight)*100%) per 10 μg/m^3 increase in air pollution exposure. Models are adjusted for gestational age at delivery.
